# Field evidence for microplastic interactions in marine benthic invertebrates

**DOI:** 10.1038/s41598-021-00292-9

**Published:** 2021-10-22

**Authors:** Stefania Vecchi, Jessica Bianchi, Massimiliano Scalici, Fabrizio Fabroni, Paolo Tomassetti

**Affiliations:** 1 Department of Sciences, University of Roma Tre, Viale G. Marconi 446, 00146 Rome, Italy; 2 Department of Ecology and Biology, University of Tuscia, Via S. Camillo de Lellis 44, 01100 Viterbo, VT Italy; 3 ARPACAL, Regional Agency for Environmental Protection, Zona Industriale 1, 89900 Vibo Valentia, VV Italy; 4 Italian National Institute for Environmental Protection and Research, ISPRA, Via di Castel Romano 100, 00144 Rome, RM Italy

**Keywords:** Ecology, Environmental sciences

## Abstract

Microplastics represent an important issue of concern for marine ecosystems worldwide, and closed seas, such as the Mediterranean, are among the most affected by this increasing threat. These pollutants accumulate in large quantities in benthic environments causing detrimental effects on diverse biocenoses. The main focus of this study is on the ‘polychaetes-microplastics’ interactions, particularly on two species of benthic polychaetes with different ecology and feeding strategies: the sessile and filter feeder *Sabella spallanzanii* (Gmelin, 1791) and the vagile carnivorous *Hermodice carunculata* (Pallas, 1766). Since not standardized protocols are proposed in literature to date, we compared efficiencies of diverse common procedures suitable for digesting organic matter of polychaetes. After the definition of an efficient digestion protocol for microplastics extraction for both polychaetes, our results showed high microplastics ingestion in both species. Microplastics were found in 42% of individuals *of S. spallanzanii*, with a mean of 1 (± 1.62) microplastics per individual, in almost all individuals of *H. carunculata* (93%), with a mean of 3.35 (± 2.60). These significant differences emerged between *S. spallanzanii* and *H. carunculata*, is probably due to the diverse feeding strategies. The susceptibility to this pollutant makes these species good bioindicators of the impact of microplastics on biota.

## Introduction

Since 1950s, plastic was produced and utilized more and more frequently^1^ and, to date, this material is the biggest polluting anthropogenic debris in marine environment^2^. On the sea’s surface float 5 trillion of plastic particles, weighing more than 260,000 tons^3^. Due to its physical–chemical properties and environmental characteristics, plastic degrades very slowly and it can persist in marine environment for thousands of years^4^.

Hydrodynamic and meteorological factors (e. g. sea current velocity, density of water mass, temperature and wind), anthropic factors (e. g. urban and industrial activities) and physical–chemical properties of plastics (shape, density, size, chemical composition) can influence their transport dynamics and accumulation in marine environment^5^.

The plastic accumulation within the Mediterranean Sea basin is a particularly intense process, so that our sea is considered one of the most plastic-polluted areas worldwide, and exactly the sixth great accumulation zone of marine litter^6^. The main factors driving this pollution are the enclosed structure of basin, the scarce outflow of surface waters, the high population density along coastlines, and the intensive fishing, tourism, shipping and industrial activities^7^.

Plastics are on the ocean’s surface, along the water column and in seafloor worldwide^8^, and all over the world benthic environments are considered sinks for microplastics as well. Benthos accumulate plastics in considerable quantities, putting at risk the health of benthic communities^9^. Plastic affecting marine environments can be categorized according to different size classes: mega (> 1 m diameter), macro (between 2.5 cm and 1 m), meso (between 5 mm and 2.5 cm), micro (between 1 μm and 5 mm) and nano (< 0.1 μm)^10^.

Microplastics (MPs), due to their small size, are a threat to the aquatic biota because MPs can be ingested by a wide range of marine organisms. Indeed, several studies demonstrated that many marine species, both vertebrates and invertebrates, to different trophic levels and with various feeding strategies ingest microplastics, such as marine birds, mammals, reptiles, fish and diverse invertebrates^11–16^. Ingestion can take place directly, accidentally assimilating throughout filter-feeding or deposit-feeding or confusing for food, indirectly, by ingesting prey of lower trophic levels which contain microplastics^17^. This event can bring to different biological and ecological effect, as internal physical and chemical damage, trophic transfer and biomagnification, absorption to microplastics surfaces of other contaminants such as heavy metal, antibiotics, polycyclic aromatic hydrocarbons and polychlorinated bisphenols, transport of pathogens and alien species that colonize microplastic surface^18^.

Several studies have highlighted differences in microplastics ingestion between feeding models, in fact, some studies show a highest microplastic concentration in suspension feeders^13,19^ while others show a greater microplastic concentration in deposit-feeders or predator as compared to other feeding strategies^12,20^.

Coastal marine invertebrates are potentially affected by coastal pollutants, such as plastic debris, and their interactions could happen at various ecological levels. Among marine invertebrates, polychaete are good indicators and they are useful for detecting environmental alterations due to their response to cumulative factors of natural or anthropogenic origin. Polychaetes are widely distributed, and, in the benthic domain, they constitute 35–70% of macroinvertebrate populations^21^. Polychaetes present all the feeding schemes that can be found among benthic organisms, omnivores, herbivores, carnivores, filter feeders, surface deposit feeder and burrowers, so they can uptake MPs under several trophic ways.

Several studies have been conducted on the MPs ingestion by polychaetes in various parts of the world^20,25,44,45^, while in the Mediterranean Sea few studies have been conducted on this. These latest studies have examined the ingestion of microfibres in annelid species of *Saccocirrus* (Bobretzky, 1872)^22^, MPs in seaworms (Muller, 1776)^23^ and MPs occurrence in six benthic invertebrate species, including the polychaete *Hediste diversicolor*^24^.

There are different methods and protocols to detect MPs within of the polychaetes, some of them involve the digestion of the whole organism^12,20,23–25^. In fact, to isolate MPs present in marine organisms can be challenging because the plastic may be hidden by biological materials, therefore most procedures involve the digestion of organic matter^26^.

Considering the importance of this environmental issue, the first aim of the present study is to assess the interaction between MPs and benthic organisms naturally exposed through the study of ingestion in two species of benthic polychaetes with different ecological features.

In this study we have preliminarily selected the most suitable environmental indicators of this type of pressure among the polychaete species present along the Italian coasts. We then analysed all the methods of study and analysis of MPs in marine organisms to choose the one that, better than others, suited the purposes of the research. The weighted analysis of the ecological characteristics and study of the polychaete species present along the Italian coasts led to the choice of the Mediterranean fanworm *Sabella spallanzanii* (Gmelin 1791) and the bearded fireworm *Hermodice carunculata* (Pallas, 1766).

For this purpose, considering the unavailability of well-established methods, the other study aim is also define a protocol to extraction of MPs by the organisms to make the appropriate assessments. The results of the present contribute to give detailed information about MP contamination level of polychaetes and offer a well-established MP detection method in marine invertebrates.

## Results

### Different protocols comparison and Method adjustment

The digestion efficiency obtained with KOH at 60 °C for 24 h (Protocol 1) was 90%, for the protocol 2 (H_2_O_2_ at 50 °C for 12 h) was 96% and finally, for the treatment with KOH at 10% at room temperature for 2–3 weeks (protocol 3) was 96%.

**Figure 1:**
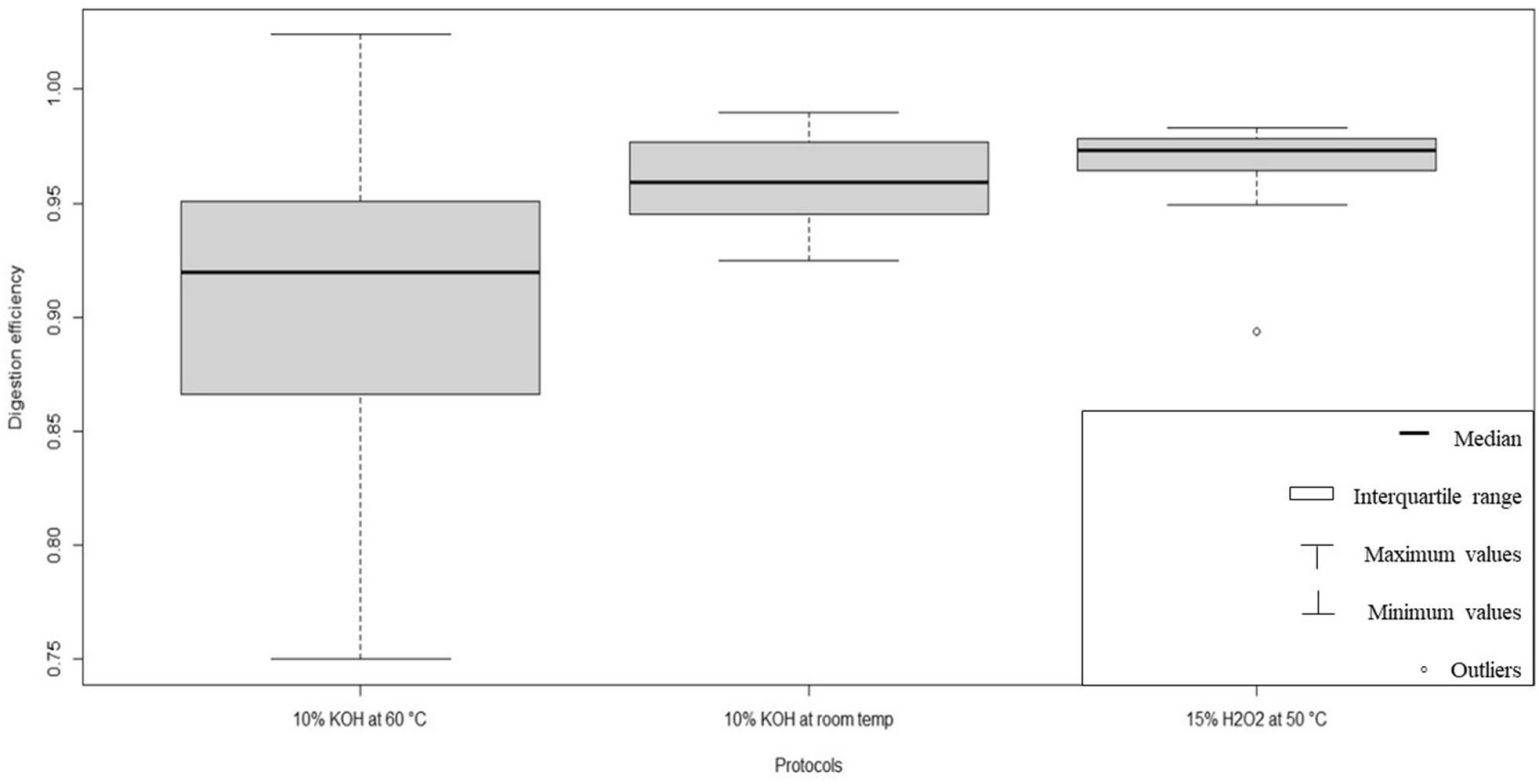
Protocol selection in *Sabella spallanzanii.* Box and whiskers plot of three digestion protocols (10% KOH at 60 °C, 10% KOH at room temp and 15% H_2_O_2_ at 50 °C, respectively from left to right protocols 1, 2 and 3). Protocols with a digestion efficiency greater than 95% are accepted. Protocols with more robust data are those with a narrow interquartile range. The figure is obtained by the software R (ver. 4.5 14,597.0, https://cloud.r-project.org).

Protocols 2 and 3 showed an acceptable digestion efficiency (> 95%). The Protocol 1, with a digestion efficiency < 95%, was not satisfactory and showed a wide variability compared to the other two protocols as the maximum and minimum values differ considerably from the central value (Fig. 1).

This variability was confirmed by the Levene test (modified robust Brown-Forsy the Levene-type) based on the absolute deviations from the median. This test showed the non-homogeneity of the variances (*p* < 0.05) (Table 1). Hence protocol 1 was discarded due to both low digestion efficiency and high variance.

**Table 1:** Statistical analysis with Leven test (B-F) and Wilcoxon rank sum test (W) and p-value of all protocols comparations in *Sabella spallanzanii* samples.

Protocols’ comparisons	Statistical analysis	Test and *p* value
a) Digestion efficiency comparation (protocols 1, 2 and 3)	Modified robust Brown-Forsythe Levene-type test based on the absolute deviations from the median	B–F = 6.5043, *p* = 0.004947
b) Digestion efficiency comparation (protocols 2 and 3)	Wilcoxon rank sum test	W = 64, *p* = 0.315
c) Membrane clogging comparation (protocol 2and 3)	Wilcoxon rank sum test	W = 44, *p* = 0.6842

A *p* < 0.05 in the B-F test (a) indicate a non-homogeneity of the variances. A *p* > 0.05 in the W test (b–c) indicate any significant difference between protocols. The statistical analysis are obtained by the software R (ver. 4.5 14,597.0, https://cloud.r-project.org).

Wilcoxon test was performed on the digestion efficiencies of protocols 2 and 3. The test showed no significant difference between the two protocols (*p* > 0.05) (Table 1). Therefore, for these two protocols we also considered the differences in membrane clogging.

The Wilcoxon test did not reveal any significant differences between the two tested protocols (*p* > 0.05) for membrane clogging (Table 1).

As both tests did not show significant differences between the two protocols, we conclude that they are equally efficient.

Qualitative considerations were made to choose the protocol to be applied to the polychaete samples. In the samples treated with 15% H_2_O_2_ a foam layer was observed not only during the digestion step, but also on the filters during filtration step. For this reason, subsequent optical examination of MPs proved particularly complicated. These problems were not found in the samples treated with 10% KOH.

For these reasons, protocol 3 was chosen for the analysis of real samples.

For *H. carunculata* additional evidences required an adjustment of the analytical approach. In fact, the numerous chaetes present in this species have not been completely digested by potassium hydroxide.

**Figure 2:**
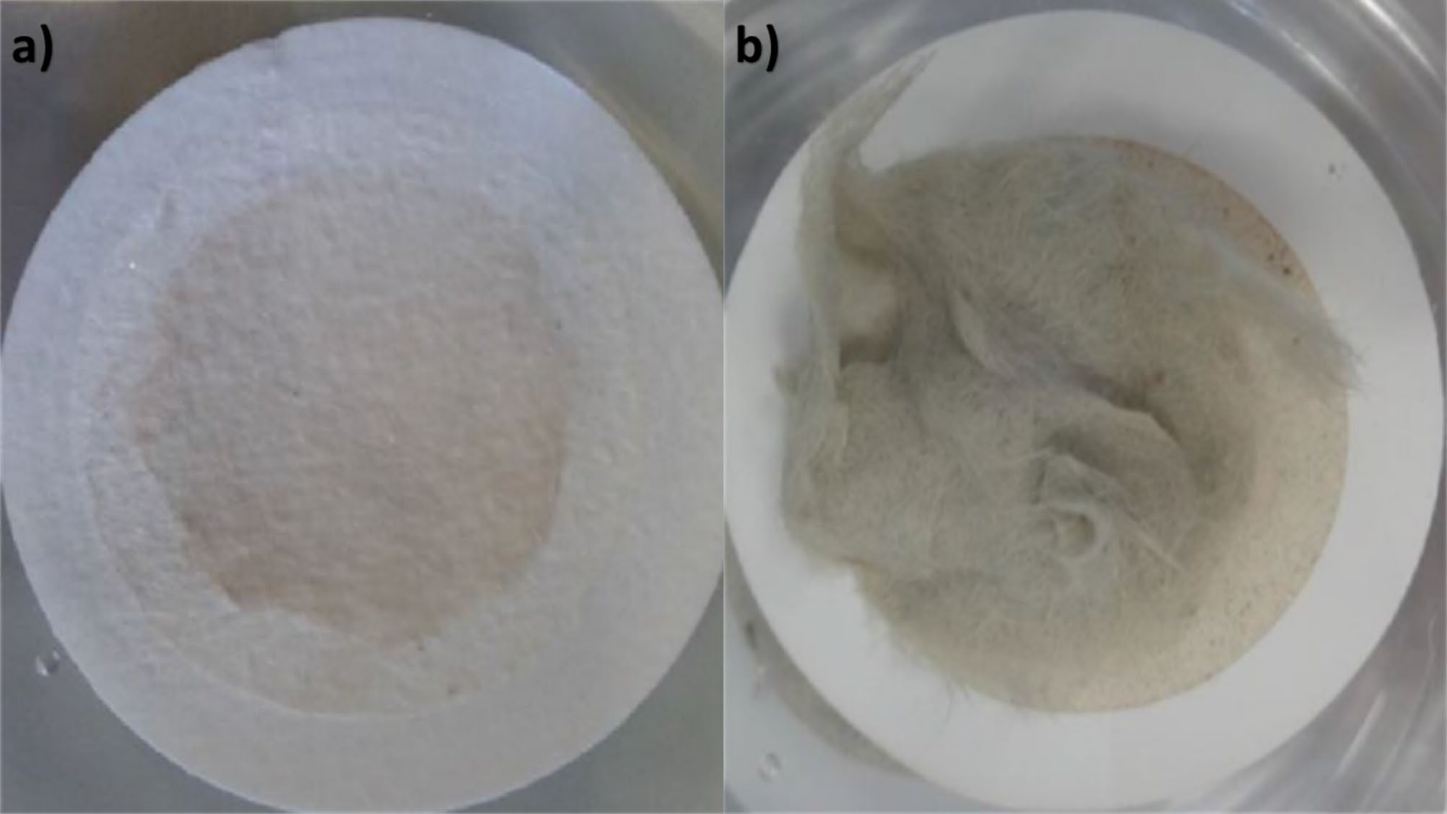
Filter before (**a**) and after (**b**) treatment with HNO_3_ and H_2_O_2_ mixture to dissolve the chaetes of *Hermodice carunculata*.

Since the chaetes are calcareous, a HNO_3_ at 5% e di H_2_O_2_ at 15% mixture was chosen to dissolve them. The results showed an almost complete digestion of the chaetes (Fig. 2).

### Microplastics in field collected samples

#### Microplastics have been found in both species

Fibers were found in great quantity but were excluded from the analysis. Only fragments and films were considered.

In total, 52 items were observed in *S. spallanzanii* samples, with a mean (± SE) of 1 (± 1.62) microplastics per individual (0.1 ± 0.2 particles/gram of tissue); 22 out of 52 individuals in total ingested MPs (FO = 42%).

Instead, 184 items were observed in *H. carunculata* samples, with a mean (± se) of 3.35 (± 2.60) microplastics per individual (0.8 ± 1.0 particles/gram of tissue). Most samples have ingested MPs, 51 out of 55 individuals (FO = 91%).

MPs ingestion significantly differed in the abundance and frequency of occurrence, in particular more MPs have been found in *H. carunculata* than in *S. spallanzanii* (Table 2).

**Table 2:** Estimates, standard error (S.E.), z-value and *p*-values with significant code (*) of Generalized Linear Models (GLM) analysis to assess differences in microplastics ingested by *Sabella spallanzanii* and *Hermodice carunculata.*

Species	Frequency of occurrence					Abundance				
	Estimate	S.E	z value	Pr ( >|z|)	Signif. codes	Estimate	S.E	z value	Pr ( >|z|)	Signif. codes
*Intercept*	2.3026	0.4690	4.909	9.15e−07	***	1.30291	0.09027	14.434	< 2e−16	***
*Sabella spallanzanii*	− 2.6127	0.5466	− 4.780	1.75e−06	***	− 0.44271	0.18315	− 2.417	0.0156	*

^1^Intercept = *Hermodice carunculata.*

Significance codes: *p < 0.05; **p < 0.01; ***p < 0.001. All the frequency of occurrence and abundance value are obtained by the software R (ver. 4.5 14,597.0, https://cloud.r-project.org).

**Figure 3:**
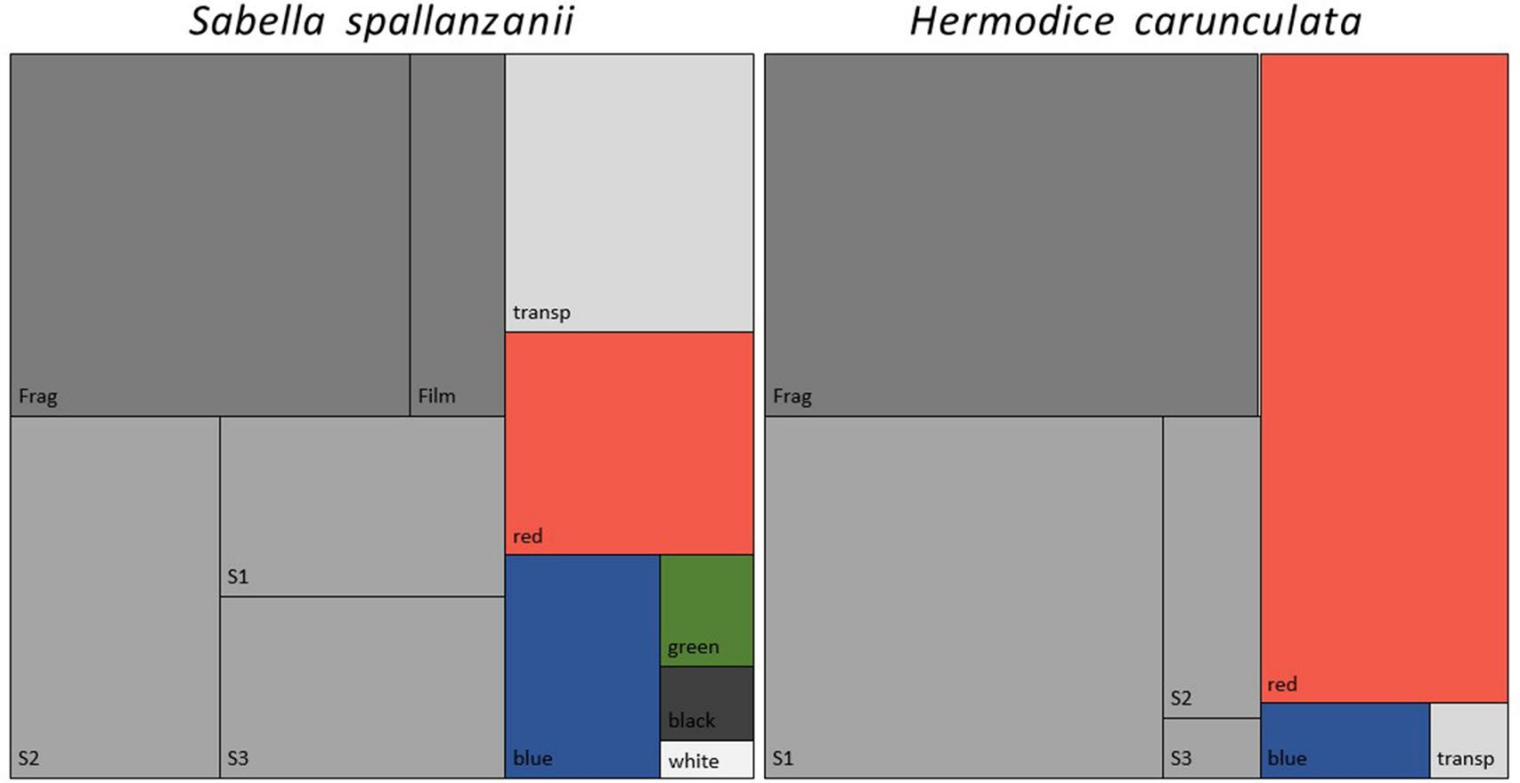
Physical characterization of ingested microplastics by *Sabella spallanzanii* and *Hermodice carunculata.* A treemap chart (Excel) with shape categories (Frag: fragments; Film: films), size classes (S1: size class 1, 90 µm–330; S2: size class 2, 330 µm–1 mm; S3: size class 3, 1–5 mm) and colour (transp: transparent; red: red; blue; green: green; black: black; white: white). The image is obtained by the software Excel (ver. 2014, https://www.microsoft.com/it-it/microsoft-365/excel) and the software PowerPoint (ver. 2014, https://www.microsoft.com/it-it/microsoft-365/powerpoint).

In *S. spallanzanii* the fragments were found in large quantities (81%) followed by films (19%) (Fig. 3). Various colors were observed: transparent items (38%), red/pink (31%), blue (19%), green (6%), black (4%) and white (2%) (Fig. 3). According to size classes, 22 MPs were found in the class 2 (42%), 15 in the class 1 and in the class 3 (29%) (Fig. 3).

In *H. carunculata* the fragments were the dominant MPs shape (99%), only one film was found (1%) (Fig. 3). Three colors were observed: red/pink items (90%), blue (7%), transparent (3%) (Fig. 3). In all *H. carunculata* samples the items of the red/pink category are pink. As for the size classes, 148 MPs were found in the class 1 (81%), 30 in the class 2 (16%) and 6 in the class 3 (3%) (Fig. 3).

**Figure 4:**
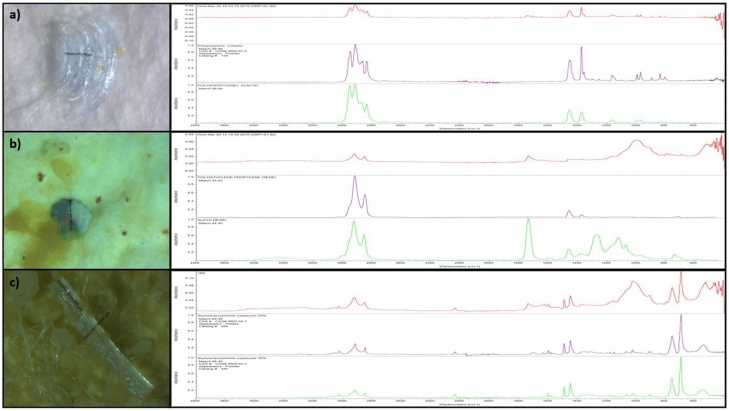
Chemical characterization of ingested microplastics by *Sabella spallanzanii* and *Hermodice carunculata*. Picture and infrared spectra of three items are reported. (**a**) polypropylene (PP) fragment found in *Sabella spallanzanii*. (**b**) mix polyethylene-polypropylene (mix PE-PP) fragment found in *Sabella spallanzanii*. (**c**) polystyrene (PS) fragment found in *Sabella spallanzanii*. The pictures are obtained by the software of image for microscope ZEN 2011 SP1 blue edition (6.1.7601) and the FT-IR spectra are obtained by the software OMNIC 9.8.286 (Thermo Fisher Scientific Inc.).

FT-IR identification confirmed that all isolated particles larger than 330 µm were plastic polymers (x = 52). Among the analyzed particles in *S. spallanzanii* 64% were made of polyethylene (PE), 15% of polystyrene (PS), 9% of polypropylene (PP) and 6% of polytetrafluoroethylene (PTFE) and a mix of polyethylene and polypropylene (mix PE-PP) (Fig. 3). In *H. caunculata* 84% were made of PE, 11% PS and 5% NY (Fig. 4).

## Discussion

Techniques used to identify MPs in fish and invertebrates have been adapted from previous studies on large vertebrates (e.g., marine mammals or seabirds)^26^. As research on this issue advances, it is essential to implement knowledge with increasingly relevant methods. Because of the difficulties of isolation and identification microplastics and the different physiology of taxonomic groups under consideration, a degree of flexibility and innovation is clearly required.

Since there are few studies that use digestion methods applied to polychaetes, in this research are used protocols (tested to study the ingestion of microplastics in the fishes) to digest organic matter of the polychaetes.

Among the digestion methods utilized we chose the most common in literature, that are a 10% potassium hydroxide (KOH) solution^27,28^ and hydrogen peroxide (H_2_O_2_)^29,30^ and nitric acid (HNO_3_)^12,31,32^, but the latter, it is advisable to use it at low concentrations, because it could corrode microplastics we are looking for. The choice of chemical treatment depends on how well it digests the organic matter and its corrosiveness towards plastic polymers.

Therefore, we excluded protocols with high concentration such as 69% of HNO_3_, because at this concentration it is certainly corrosive even to microplastics inside the organism^32^, or protocols that use NaOH because it is a less effective digestive agent than others and, moreover, it degrades some plastic polymers^33,34^.

The results, obtained from the comparison among the three protocols, led to the final choice, that is the Foekema protocol^27^ (10% KOH solution at room temperature for 2–3 weeks) as defined treatment. Potassium hydroxide is a widely used reagent in the alkaline digestion of the organic matter for the extraction of microplastics from biota, in polychaetes, in other invertebrates and in gastrointestinal tract of the vertebrates^27,28,34–36^.

The Foekema protocol applied to *S. spallanzanii* is used for *H. carunculata* samples, but these have a lot of chaetes that during the procedure are not completely digested. So, it was necessary to perform a second digestion for the only chaetes of this species. For this phase we have applied a new protocol tested in an article that highlighted how different chemical reason implicated in the digestion have a different effect depending on matter to digest^32^. In particular, oxidative reaction, with reagent as hydrogen peroxide and nitric acid at low concentrations, are recommended for hard substrate such as chitinous exoskeletons and carbonate shells^32^. Since *H. carunculata* chaetes are calcareous the protocol developed in this article is applied. As expected, the mixture (5% HNO_3_ + 15% H_2_O_2_) digested the chaetes efficiently.

In addition to the experimental approach, this work provides data on the presence and characteristics (shape, size, color, polymer) of microplastics in benthic polychaetes with different feeding models.

Studies on microplastics ingestion in polychaetes have already been conducted in different parts of the world^12,13,20,22,25,37–45^.

Instead, in Mediterranean Sea there are few studies concerning that subject^23,24^. This work is the first to investigate the presence of microplastics in the polychaetes *S. spallanzanii* and *H. carunculata* from their natural habitat, in the Mediterranean Sea.

The current study shows the highest concentrations and frequency of occurrence in *H. carunculata* compared to *S. spallanzanii.* Furthermore, in both polychaetes there were fragment-shaped microplastic particles, made of polyethylene and of small size (< 1 mm) in greater quantity. On the other hand, a difference emerged in the type of color of microplastics founded in the two species (Fig. 3).

This study showed an interaction between microplastics and polychaetes. At the beginning of the study, both species were selected for their biology and ecology and especially for their feeding strategy, as well as for their easy findability. Our results demonstrated that these polychaetes are subjected to this anthropic impact. In particular, concerning the feeding strategy, the data showed highest values in *S. spallanzanii* compared to *H. carunculata,* both in terms of abundance and frequency of occurrence.

These two species have a different food strategy: the fanworm is a filter organism, while the green fireworm is a predatory carnivore and deposit-feeders. Feeding behavior and strategy greatly influence the ingestion rate of plastic particles. The results of some studies, although contrasting with each other, show clearly how the quantities of ingested microplastics vary according to the food models^13,19,20,39^. Due to this discrepancy among the results, further studies on microplastics ingestion should be investigated, possibly in field conditions.

From the results obtained in the current study we can assume that filter feeders are less exposed to microplastics than deposit-feeders or carnivorous. In particular, filter-feeders could be more selective towards food particles compared to other organisms, but another explanation could be a different concentration of microplastics between the two sampling areas or a greater availably of microplastics on the sediment surface compared to the water column. The fact that more microplastics are ingested to predator *H. carunculata* could also means that these particles are more easily transferred between species, along feeding web (biomagnification), although a deliberate ingestion of microplastics, confusing for a food particle, cannot be excluded.

Comparing our data with data obtained in a study conducted on the polychaete *Hediste diversicolor*, sampled in the Adriatic Sea, differences emerged, that is only one microplastic was detected of a total of 100 individuals^24^ (Table 3a). In another study conducted in Mediterranean Sea, along the Tunisian coast, the abundance of microplastics varied (from 0.5 particles/gram to 3.7 particles/gram of tissue) depending on the sampled site and our data is closer to those sites where the concentration of microplastics was lower^23^ (Table 3b).

**Table 3:** Some studies that investigate the presence of microplastics (Mps) in polychaetes in different parts of the world.

Location	Species	Mps/individual or gram	Authors
Adriatic Sea	*Hediste diversicolor*	Only 1 mps in 100 individuals	(a) Piarulli et al*.* (2020)
Tunisian coast of Mediterranean Sea	*Hediste diversicolor*	From 0.5 to 3.7 mps/gram	(b) Missawi et al*.* (2020)
North Sea and Barents Sea	*Galathowenia oculata*	0.78 ± 0.59 mps/individual	(c) Knutsen et al*.* (2020)
	*Galathowenia fragili*		
	*Owenia borealis*		
Oslofjord	*Hediste diversicolor*	1–2 mps/individual	(d) Bour et al*.* (2018)
	*Sabella pavonina*		
French, Belgian and Dutch coasts in the North Sea	*Arenicola marina*	1.2 ± 2.8 mps/gram	(e) Van Cauwenberghe et al*.* (2015)

The results in our samples are similar to these of other polychaetes species outside the Mediterranean. Levels of microplastics have been reported in some polychaetes as *Galathowenia oculata* (Zachs, 1923), *Galathowenia fragili* (Nilsen & Holthe, 1985) and *Owenia borealis* (Koh, Bhaud & Jirkov, 2003) sampled in North Sea and Barents Sea, with an overall abundance of 0.78 ± 0.59 particles/individuals, although microplastics concentrations are higher in the tube than in the tissue^25^ (Table 3c). In another studies, 1–2 particles/individuals were estimated in *Hediste diversicolor* and *Sabella pavonina* (Savigny, 1822), from the inner Oslofjord in Norway^20^ (Table 3d), whereas in the individuals of *Arenicola marina* (Linnaeus, 1758) collected along the French, Belgian and Dutch coasts in the North Sea, has been founded an average of 1.2 ± 2.8 microplastics/gram of tissue^12^ (Table 3e). Instead, in the fecal contents of polychaetes collected along the coasts in South Korea, has been reported a mean of 0.71 ± 1 microplastics / gram of tissue^45^. It should be considered that some of the studies mentioned above also evaluated fibers and therefore our results could differ.

However, different extraction methods are utilized to study microplastics presence and characterization in the marine organisms, and this not always allow a correct comparison with species of other studies. For this reason, standardized extraction methods are particularly important.

In this study we have chosen to exclude fibers from the analysis to avoid the overestimation of data, given the large quantity of fibers found. Despite our attention during all the laboratory processes, the possibility of environmental contamination is not erased. Since fibers are ubiquitous^46^, it is not possible to exclude environmental contamination during the sampling phases.

The fragments were found in dominant form in both polychaetes. After fibers, fragments are the most frequent type of microplastic in many invertebrates^20,23,30,47^.

Our results show that the most of ingested microplastics in *S. spallanzanii* are transparent. Particles of this color are commonly found in organisms but are not predominant^20,48,49^. Instead, *H. carunculata* ingested pink microplastic particles in larger quantity, an unusual color in this type of studies. These particles could derive from the fragmentation of anthropogenic debris poured into the coastal environment but also from initially red particles that turn pink after chemical or environmental degradation.

In *S. spallanzanii* particles ranging in size from 330 µm to 1 mm (class 2) were in greater concentration, whereas in *H. carunculata* particles ranging in size from 90 to 330 µm (class 1) were found in large quantities. Microplastics can be confused for natural preys or ingested directly during feeding behavior due to their similar size^50^. Size selectivity has been observed in different species as oligochaetes^51,52^, decapod and euphausiid^11^.

Polymer identification is important as different polymers can have varying impacts on the marine ecosystem. Consistent with other studies^20,23^ our result show that most of ingested microplastics are made of polyethylene (PE), in both polychaetes. Although polyethylene is a low-density polymer, through the biofouling process and atmospheric agents, it sinks and becomes accessible to the benthic organisms^53^. This data could indicate the expansive use of plastic items made of polyethylene such as plastic bags, food packaging, trays and containers, various bottles, houseware. Polyethylene is the most produced plastic polymer^54^. Coastal areas are subjected to a high anthropogenic pressure, caused by the proximity of the urban areas, rivers, sewage and fishing activities, which makes them the most polluted areas by microplastics.

## Conclusion

In the Mediterranean Sea there is a lack of widely accepted protocols to assess pollution by MPs in marine invertebrates. MPs are widespread in our seas and develop efficient protocols is important for monitoring and managing the impact of these marine litter on biodiversity.

Our study allowed to define an efficient digestion protocol for MPs extraction from two polychaetes species, *S. spallanzanii* and *H. carunculata,* in the Mediterranean Sea.

These two species of polychaetes were used for the first time to assess the impact of MP pollution. With this study the presence of MPs in *S. spallanzanii* and *H. carunculata* were verified and consequently their susceptibility to this type of pollution. Therefore, both polychaetes can be considered good bioindicators of the potential impact of MPs on biota and a useful tool for the monitoring of the coastal marine environments of the Mediterranean Sea.

Finally, the difference in MPs ingestion between species with different feeding strategies is very important and should be further investigated, possibly by conducting field studies and therefore considering all the interactions that occur naturally between organisms and the environment in which they are immersed.

## Methods

### Sampling and species

The species selection was made considering some ecological and practical aspects, including habitats, distribution, feeding strategy, species availability and sampling feasibility. Considering that the seafloors are areas with high accumulation of MPs^9^, we focused on benthic polychaetes since they are ubiquitous, they occupy practically all marine ecological niches, have different trophic strategies, and can be sampled easily.

**Figure 5:**
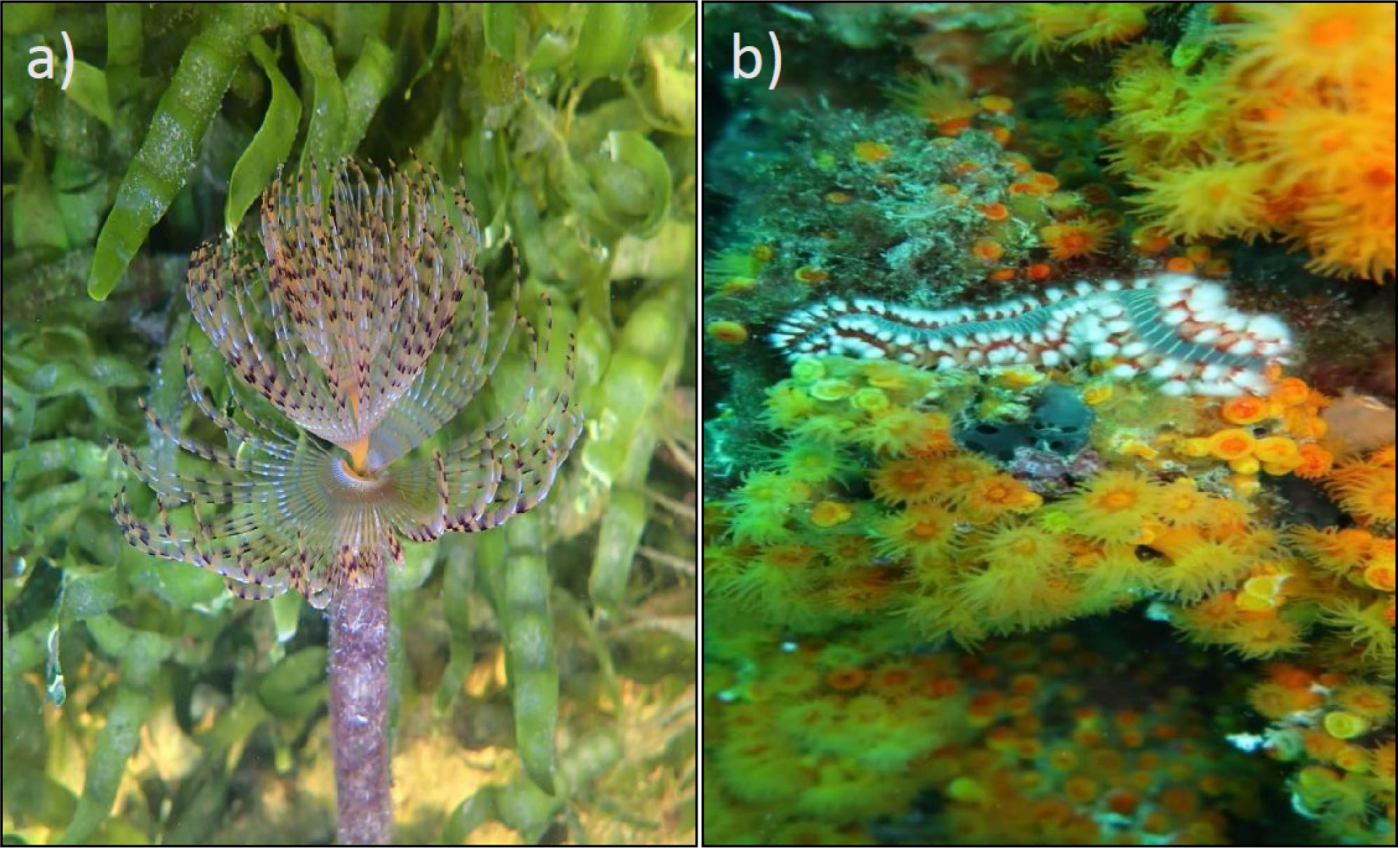
The investigated biological model: *Sabella spallanzanii* (A) and *Hermodice carunculata* (B). The photo images were taken by Paolo Tomassetti (**a**) and Fabrizio Fabroni (**b**).

To investigate MPs ingestion changes, depending on feeding strategy^13^, we selected two species with different feeding guilds: a suspension filter feeder, *S. spallanzanii* (Fig. 5a) and a predator, *H. carunculata* (Pallas, 1766) (Fig. 5b). Furthermore, both species were chosen based upon: their high density on coastal waters; wide distribution in Mediterranean Sea; the presence in various habitats; and affordable sampling effort; commonly found in environments known to have high densities of MPs.

The Mediterranean fanworm, *S. spallanzanii*, is a sessile benthic polychaete belonging to Sabellidae family^55^. *S. spallanzanii* live in a membranaceous tube attached to the hard substrates (rocks, artificial materials and benthic organisms) in the shallow subtidal zones, at a depth range of 1–30 m^56^. The species is a filter feeder that feeds on suspension particles as a dissolved organic matter in the water column and plankton^57^. Furthermore, this polychaete is able to filter large quantities of water, approximately 12 m^3^ per day^58^.

The bearded fireworm, *H carunculata*, its vernacular name derives from the tufts of white, sharp, and venomous chaetae, is an errant benthic polychaete belonging to Amphinomidae family^55^. *H. carunculata* inhabits shallow water, including the intertidal zone and is common in a wide variety of natural and artificial habitats^59^. The bearded fireworm is a predator and a scavenger of sessile and slowly moving organisms, especially cnidarians, or injured prey and carrion, but it also feeds frequently on algae and detritus remains^60^.

The choice of sampling sites was made considering two parameters: the feasibility of sampling the selected species in an appropriate number for their study and the selection of coastal areas in which it was assumed that the interaction between the selected species and the MPs dispersed in the marine environment was maximum. For this reason, were selected sites with high concentrations of MPs, that is harbours and coastal areas^61,62^.

In particular, *S. spallanzanii* has been sampled in harbours, where the species is particularly prevalent and grows on the vertical wall of the quay. *H. carunculata*, on the other hand, has been sampled along coastal areas, where the species is very frequent especially along the southern coasts of Italy.

Sampling was carried out from October 2018 to August 2019. A total of 108 specimens (52 *S. spallanzanii* and 55 *H. carunculata*) were collected. The sampling areas are located in three Italian regions of the Western Mediterranean Sea.

**Figure 6:**
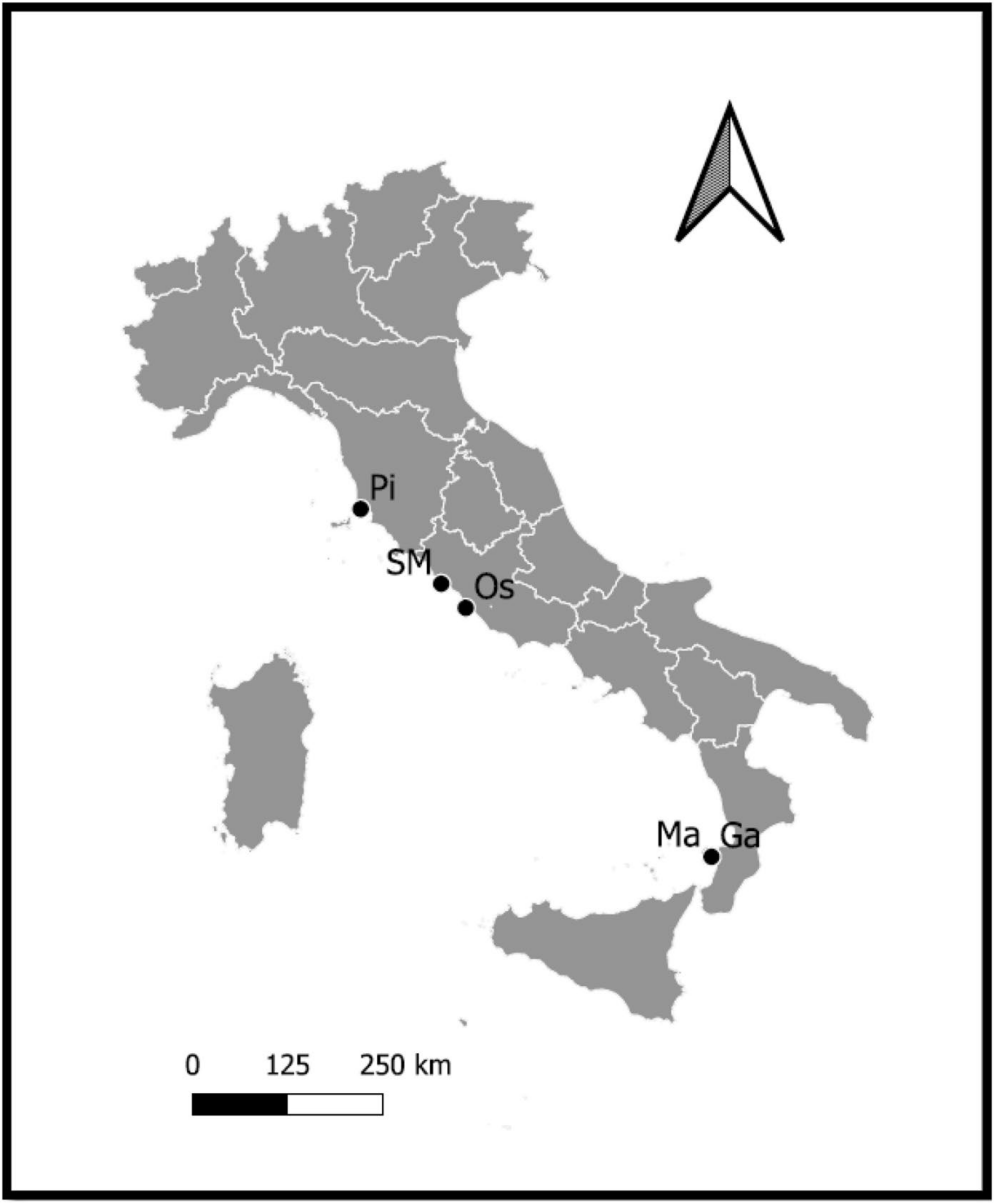
Location of the sampling sites of the Mediterranean fanworm *Sabella spallanzanii* (Os, SM, Pi) and the bearded fireworm *Hermodice carunculata* (Ga and Ma). In alphabetical order: Ga = Scoglio della Galea; Ma = Secca del Mantineo; OS = Ostia; Pi = Piombino; SM = Santa Marinella. The figure is obtained by the QGIS software (ver. 3.16.7-Hannover, https://www.qgis.org/it/site/index.html).

*S. spallanzanii* samples were collected in three harbours, Ostia, Santa Marinella and Torre del Sale (Central and Northern Tyrrhenian Sea), (Fig. 6, sites Os, SM and Pi, respectively) located in Latium and Tuscany regions. The specimens were collected by hand along vertical wall of the quay.

*H. carunculata* samples were collected in two coastal areas, Secca del Mantineo and Scoglio della Galea (Southern Tyrrhenian Sea), (Fig. 6, sites Ma and Ga, respectively), in Calabria region. The specimens were collected by hand by scuba divers at depths between 10 and 20 m.

The specimens of both species were rinsed with filtered sea water, placed in specific glass jars with 70% ethyl alcohol solution as fixative.

### Different protocols comparison

*S. spallanzanii* samples were thawed at room temperature and extracted from the tubes.

The organisms were rinsed with filtered Milli-Q water, weight and length were recorded and each sample was collected in a jar to digestion treatment. We tested three different protocols:

Protocol 1) 10% potassium hydroxide (KOH) solution to 3 × the volume of the tissue, incubation at 60 °C for 12 h^28^.

Protocol 2) 15 ml of 15% hydrogen peroxide (H_2_O_2_) for each gram of tissue, incubation at 50 °C for 12 h^29^.

Protocol 3) 10% potassium hydroxide (KOH) solution to 3 × the volume of the tissue, incubation at room temperature for 2–3 weeks^27^.

Digestion was performed on the whole body of organism. To evaluate the digestion efficiency, 10 samples for each protocol were used, resulting a total of 30 organisms.

Once the organic material was degraded, the jars content was filtered through glass microfiber membranes (Whatmann GF/F™: 0.7 μm pore size) using a vacuum pump system.

Before and after filtration, the membranes were dried at 40 °C for 24 h and weighed on a scale (Mettler) with 0.01 mg precision.

To evaluate digestion efficiency (%) the following formula^63^ was used:

$$Digestion\,\, efficiency \left( \% \right) = \frac{{Wi - \left( {Wa - Wb} \right)}}{Wi} \times 100$$

where Wi = Initial weight of biological materials; Wa = Weight of dry membrane after filtration and Wb = Weight of dry membrane before filtration.

Membrane clogging (filters g-1) was calculated using the following formula^32^:

$$Membrane\,clogging \left( {filters \cdot g^{ - 1} } \right)\, = \,\frac{N}{{Wi - \left( {Wa - Wb} \right)}}$$

where N = number of membranes required; *Wi* = Initial weight of biological materials; *Wa* = Weight of dry membrane after filtration and *Wb* = Weight of dry membrane before filtration.

Levene's Test of Equality of Variances was applicated to compare the digestion efficiencies of the three protocols. After the homogeneity test, the digestion efficiencies and the membrane clogging were compared performing nonparametric method, the Wilcoxon rank sum test.

### Method adjustment

*H. carunculata* samples were rinsed with Milli-Q water to remove ethyl alcohol. Weight and length were recorded, and each sample was collected in a jar to digestion treatment.

*H. carunculata* samples were digested with the selected protocol for *S. spallanzanii* and later subjected to a further procedure due to the presence of many chaetes.

Since the chaetes of this species are calcareous an oxidizing agent was used. For each sample, the chaetes were taken and placed in a jar. A 5% HNO_3_ + 15% H_2_O_2_ mixture^32^ to 3 × the volume was added. The jars were incubated at 40 °C for overnight and the solution obtained filtered through glass microfiber membranes (Whatmann GF/C™: 1.2 μm pore size) using a vacuum pump system.

### Microplastics in field collected samples

*S. spallanzanii* samples were digested with the selected procedure (protocol 3), *H. carunculata* samples were processed with the protocol developed in “method adjustment”.

Ingested plastic items were quantified and subjected to physical and chemical characterization.

Filters were visually analysed using a stereomicroscope (MARCA) and plastic particles were identified following the MEDSEALITTER protocol (MEDSEALITTER deliverable 4.6.1 “Final common monitoring protocol for marine litter”), considering the resistance to contact with tweezers, uneven edges, distinctive colours and those that show a dark sticky mark when touched with a hot needle^64^.

Shape type (fibre, fragment or film), colour (black, blue, green, red/pink, transparent or white) and size class (class 1: 90–330 µm, class 2: 330 µm–1 mm, class 3: 1–5 mm)^3,10^ were recorded for each identified plastic particles.

Fourier Transform Infrared spectrophotometer with Attenuated Total Reflection (ATR-FTIR, Thermo Fisher Scientific, Madison, WI, USA) was used to characterize polymers of items larger than 300 µm.

### Contamination control

During all lab procedures a Tyvek protective suit was used and samples were processed under a laminar flow cabinet to avoid secondary contamination. Furthermore, all laboratory instruments and tools were washed with ultrapure water and checked under a stereomicroscope, to prevent cross-contamination. Procedural blanks were used in all steps (digestion, filtration and identification) for each batch of processed samples. No airborne or reagent contamination was recorded.

### Statistical analysis

The difference in the frequency of occurrence and abundance of the ingested MPs between the two species, was analysed using Generalized Linear Models (GLM).

The null hypothesis is that the frequency of occurrence and abundance in the two species are equal, therefore MPs ingested amount in *S. spallanzanii* is the same in *H. carunculata*. The occurrence frequency data were analysed using binomial distribution. Instead, abundance was analysed using the negative binomial distribution, due to data overdispersion.

All statistical analysis were performed with the software R 3.5.3^65^, using “lawstat”^66^, “mass”^67^ and “visreg”^68^ packages. The significance level was established to 5%.
